# Relationships Between Reproductive History and Mortality From Cardiovascular Diseases Among Japanese Women: The Japan Collaborative Cohort Study for Evaluation of Cancer Risk (JACC) Study

**DOI:** 10.2188/jea.JE20190020

**Published:** 2020-11-05

**Authors:** Kanami Tanigawa, Satoyo Ikehara, Takashi Kimura, Hironori Imano, Isao Muraki, Kokoro Shirai, Akiko Tamakoshi, Hiroyasu Iso

**Affiliations:** 1 Public Health, Department of Social Medicine, Osaka University Graduate School of Medicine, Osaka, Japan; 2 Faculty of Medicine, Hokkaido University, Hokkaido, Japan; 3 Department of Public Health Medicine, Faculty of Medicine, University of Tsukuba, Ibaraki, Japan

**Keywords:** maternal age, parity, cardiovascular diseases, mortality, prospective studies

## Abstract

**Background:**

Reproductive history has been addressed as a risk factor for cardiovascular disease (CVD). We examined the relationship between reproductive history and CVD mortality in Japanese women.

**Methods:**

We followed 53,836 women without previous CVD or cancer history from 1988–1990 to 2009 in a prospective cohort study. Hazard ratios (HRs) and 95% confidence intervals (CIs) of CVD mortality were estimated according to the number of deliveries and maternal age at first delivery.

**Results:**

During the follow-up, 2,982 CVD-related deaths were identified. There was U-shaped association between the number of deliveries and risk of CVD mortality with reference to three deliveries, although the excess risk of CVD mortality associated with ≥5 deliveries was of borderline statistical significance. The corresponding multivariable HRs were 1.33 (95% CI, 1.12–1.58) and 1.11 (95% CI, 0.99–1.24). In addition, higher CVD mortality was associated with maternal age ≥28 years at first delivery than maternal age of 24–27 years at first delivery. The multivariable HRs were 1.22 (95% CI, 1.10–1.36) for 28–31 years at first delivery and 1.26 (95% CI, 1.04–1.52) for ≥32 years at first delivery. Moreover, among women with ≥3 deliveries, maternal age ≥28 years at first delivery was associated with 1.2- to 1.5-fold increased CVD mortality.

**Conclusion:**

The number of deliveries showed a U-shaped association with risk of CVD mortality. Higher maternal age at first delivery was associated with an increased risk of CVD mortality, and excessive risk in women aged ≥28 years at first delivery was noted in those with ≥3 deliveries.

## INTRODUCTION

Cardiovascular disease (CVD) is the leading cause of death and disability in the world, and 17.9 million deaths due to CVD occurred worldwide in 2016.^1^ The latest guidelines on CVD prevention in women from the American Heart Association have indicated that reproductive history related to pregnancy should be taken account to identify high-risk women for CVD.^2^ The reproductive history related to pregnancy, such as the number of deliveries and maternal age at first delivery, is related to lifetime experiences of large hormonal changes during pregnancy, which may affect metabolic profiles during and after pregnancy and subsequent risk of CVD. However, the association between reproductive history and risk of CVD remains controversial. Some prospective cohort studies have shown a U-shaped relationship between the number of deliveries and risks of CVD incidence^3^ and CVD mortality.^4^^–^^8^ However, other studies have found positive association with risks of CVD incidence^9^^,^^10^ and CVD mortality,^11^ inverse association with risk of CVD mortality,^12^ and no association with risk of CVD mortality.^13^^,^^14^ In addition to the association with the number of deliveries, a number of prospective cohort studies have shown that early maternal age at first delivery was a risk factor for risk of CVD mortality,^4^^,^^14^^–^^16^ but other studies showed no inverse association between maternal age at first delivery and risk of CVD mortality.^15^^–^^17^ The higher maternal age at first delivery was associated with risk of mortality from subarachnoid hemorrhage^18^ and incidence of coronary heart disease (CHD).^19^

Between 1945 and 2017, total fertility rate (TFR) has rapidly decreased and maternal age at first delivery has increased in Japan. Now, Japan is one of the countries with the lowest TFR (1.43 in 2017) and the highest maternal age at first delivery (30.7 years in 2017)^20^ among Organisation for Economic Cooperation and Development countries. Previous reports in the Japan Collaborative Cohort Study for Evaluation of Cancer Risk (JACC) study showed a brief summary of the association between reproductive history and age-adjusted risk of CVD mortality during 13-year follow-up,^21^ but they did not conduct multivariable-adjusted analysis.

In the present study, we examined the association of the number of deliveries and maternal age at first delivery with mortality from CVD, stroke, and CHD among Japanese women in a nationwide prospective cohort study.

## MATERIAL AND METHODS

### Study population

The JACC Study was designed to evaluate the relationship of lifestyle and mortality from all causes, CVD, and major cancers, and to provide prevention strategies for chronic diseases. In 45 communities across Japan, we enrolled 110,585 individuals aged 40–79 years (46,395 men and 64,190 women) who completed a baseline questionnaire during 1988–1990 and were followed until the end of 2009. The details of the study are described elsewhere.^22^ Our entire study design was approved by the Ethics Committees of Hokkaido University Graduate School of Medicine and Osaka University, Japan.

**Figure 1.**
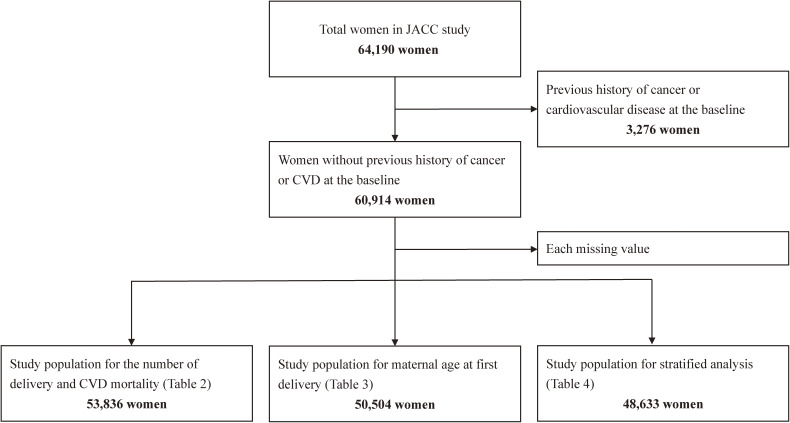
Flowchart of study exclusion and inclusion

Among 64,190 women, 3,276 women with previous history of cancer or CVD at the baseline were excluded, yielding a final sample of 60,914 women. Within this population, we analyzed the number of deliveries (*N* = 53,836) and maternal age at first delivery (*N* = 50,504) and their association with risk of CVD mortality, after excluding missing data on individual reproductive history (Figure 1).

### Baseline survey

At the baseline, a self-administered questionnaire was conducted to collect information (from all individuals) about demographic characteristics, individual health condition, lifestyle, and medical history of hypertension and diabetes, and information (from women only) about reproductive history, including the number of deliveries and maternal age at first delivery.

Reproductive history was assessed using two self-reported questions: “How many times did you experience delivery?” and “How old were you when you experienced the first delivery?”. The answers were written by the participants and were divided into six categories for the number of deliveries (0, 1, 2, 3, 4, and ≥5 deliveries), and into five categories for maternal age at first delivery (<20, 20–23, 24–27, 28–31, and ≥32 years).

### Mortality surveillance

For mortality surveillance, investigators systematically reviewed death certificates, which had been received in each municipality and collected by the Ministry of Health, Labour and Welfare through public health centers and prefectural governments. The follow-up was conducted to identify the vital status of the participants until the end of 2009, including 10 communities that ended their follow-up before 2009 (four in 1999, four in 2003, and two in 2008). We assigned the International Classification of Diseases, 10th version (ICD-10) codes I01–I99, I60–I69, and I20–I25 to mortality from CVD, stroke, and coronary heart disease (CHD), respectively.

### Statistical analysis

The person-years of follow-up were calculated as the period from the date at which the baseline questionnaire was answered through the earliest date of death, moving out, or end of follow-up, whichever came first. We calculated age-adjusted means and prevalence of cardiovascular risk factors according to the number of deliveries (0, 1, 2, 3, 4, and ≥5 deliveries) and maternal age at first delivery (<20, 20–23, 24–27, 28–31, and ≥32 years). Test for trends, which were adjusted for age at baseline, were performed according to categorized value for the number of deliveries and the median value in each category for maternal age at first delivery.

Cox proportional hazard regression models were used to calculate the age-adjusted and multivariable-adjusted hazard ratios (HRs) and their corresponding 95% confidence intervals (CIs) for each category concerning the number of deliveries and maternal age at first delivery. For both variables of reproductive history, the median category value was used as the reference value (three deliveries for number of deliveries, and 24–27 years for maternal age at first delivery). Adjustment variables were chosen primarily based on findings from previous studies as potential confounding variables.^8^^,^^16^ They were age (continuous), body mass index (BMI; quintiles), sleep duration (<5, 6, 7, 8, and ≥9 hours/day), walking habit (almost never, 0.5, 0.5–0.9, and ≥1 hours/day), exercise (almost never, 1–2, 3–4, and ≥5 hours/week), smoking status (never, past, and current smoker), alcohol intake (never, past, and current drinker), education (primary school, junior high school, high school, and college), occupation (fulltime job, part-time job, self-employed, housewife, no job, and other), marital status (married, widowed, divorced, and never married), perceived mental stress (low, moderate, and high), history of hypertension (yes and no) and history of diabetes (yes and no). Furthermore, the number of delivery was also adjusted in the analysis for association between maternal age at first delivery and CVD mortality.

Additionally, the analysis for maternal age at first delivery was stratified by the number of deliveries. In this stratified analysis, maternal age at first delivery was classified into three categories: <24, 24–27 (reference), and ≥28 years, and the number of deliveries was classified into five categories: 1, 2, 3, 4, and ≥5 deliveries.

All statistical tests were two-sided, and a *P* < 0.05 was considered statistically significant. All analyses were conducted using SAS software version 9.4 (SAS Institute, Inc., Cary, NC, USA).

## RESULTS

Table 1 shows mean age, age-adjusted mean values, and prevalence of risk factors according to the number of deliveries and maternal age at first delivery. Compared to nulliparous women, those with higher number of deliveries had higher BMI, lower educational level, and higher prevalence of hypertension. On the other hand, women who were older maternal age at first delivery had lower BMI than those who were younger maternal age at first delivery. Moreover, women who were older maternal age at first delivery had higher education level and were less likely to drink alcohol, walk regularly, and exercise. Compared to women who answered the number of delivery or maternal age at first delivery, women who did not answer had the higher prevalence of current smoking and were less likely to have spouse. Other risk factors were not different from women who answered (not shown it table).

**Table 1.** Age and age-adjusted baseline characteristics of participants according to the number of deliveries and maternal age at first delivery

	Number of deliveries (*n* = 53,836)							Maternal age at first delivery, years (*n* = 50,504)					

	0	1	2	3	4	≥5	*P* for trend	<20	20–23	24–27	28–31	≥32	*P* for trend
Number	1,928	4,025	20,260	16,879	6,168	4,576		925	16,332	24,687	6,719	1,841	
Age, year^a^	59.5	56.5	53.4	56.4	63.0	69.1	<0.01	61.4	57.9	56.2	57.9	58.0	<0.01
Age at first delivery, year^a^	—	27.7	25.2	24.5	24.0	23.2	<0.01	—	—	—	—	—	—
BMI, kg/m^2^	22.6	22.6	22.8	23.0	23.2	23.1	<0.01	23.4	23.1	22.8	22.8	22.7	<0.01
Sleep, h/day	7.2	7.1	7.1	7.1	7.1	7.3	<0.01	7.2	7.1	7.1	7.0	7.0	<0.01
Current smoker, %	9.1	8.6	4.6	3.8	4.7	6.1	<0.01	12.0	6.0	4.0	4.3	6.3	<0.01
Current drinker, %	16.9	15.9	15.0	14.3	15.1	15.4	0.09	22.1	16.7	14.2	13.4	12.3	<0.01
Walking ≥1.0 h/day, %	42.6	48.2	50.9	53.0	55.4	53.0	<0.01	54.3	54.2	51.5	48.9	48.2	<0.01
Exercising ≥5.0 h/week, %	4.1	4.5	4.3	4.3	5.6	6.0	<0.01	4.8	5.4	4.7	3.6	3.6	<0.01
College or higher education, %	14.7	11.7	11.1	9.2	7.8	6.7	<0.01	2.5	7.2	10.6	13.0	14.6	<0.01
High mental stress, %	21.9	21.9	20.1	19.4	19.3	21.7	0.09	21.8	19.4	19.7	21.0	22.9	<0.01
Having job, %	44.5	41.6	44.6	47.6	47.6	48.6	<0.01	45.0	46.2	47.0	45.1	41.7	<0.01
Having spouse, %	68.6	75.8	85.5	87.4	84.9	80.9	<0.01	74.0	84.0	86.2	86.7	84.4	<0.01
History of hypertension, %	20.3	21.3	22.3	22.6	23.2	22.8	<0.01	24.7	22.8	21.4	21.4	19.7	<0.01
History of diabetes, %	4.6	4.8	3.6	4.0	3.5	4.4	0.31	5.1	4.0	3.8	3.2	4.3	0.05
Number of deliveries, times	—	—	—	—	—	—	—	3.7	3.1	2.8	2.4	1.7	<0.01

^a^Crude mean value were shown.

During the median 19.4-year follow-up of 53,836 women aged 40–79 years, 2,982 women died from CVD, 1,312 from stroke, and 561 from CHD. The association between number of deliveries and risk of CVD mortality seemed to be U-shaped (*P* for non-linearity <0.01) (Table 2). Compared to women who experienced three deliveries, nulliparity was associated with increased risks of mortality from stroke and CVD. The multivariable HRs were 1.40 (95% CI, 1.08–1.82) for stroke and 1.33 (95% CI, 1.12–1.58) for CVD. Furthermore, extremely high number of deliveries (≥5 deliveries) was of borderline statistical significance with risks of CVD mortality when compared to three deliveries. Multivariable HRs were 1.12 (95% CI, 0.95–1.32) for stroke and 1.11 (955 CI, 0.99–1.24) for CVD.

**Table 2.** Hazard ratios and 95% confidence intervals of mortality from cardiovascular disease according to the number of deliveries among women aged 40–79 years

	Number of deliveries					

	0	1	2	3	4	≥5
Person-years	30,963	64,787	349,279	288,680	98,781	65,229
**Stroke**						
Number of cases (deaths)	80	72	259	334	246	321
Age-adjusted HR (95% CI)	1.50 (1.17–1.92)	0.95 (0.73–1.22)	0.97 (0.82–1.14)	1.00	1.09 (0.92–1.29)	1.20 (1.02–1.41)
Multivariable-adjusted HR (95% CI)^a^	1.40 (1.08–1.82)	0.92 (0.71–1.19)	0.96 (0.81–1.13)	1.00	1.05 (0.89–1.25)	1.12 (0.95–1.32)
**Coronary heart disease**						
Number of cases (deaths)	31	43	103	141	98	145
Age-adjusted HR (95% CI)	1.35 (0.91–2.00)	1.34 (0.95–1.88)	0.94 (0.73–1.22)	1.00	0.98 (0.76–1.28)	1.18 (0.92–1.52)
Multivariable-adjusted HR (95% CI)^a^	1.19 (0.79–1.79)	1.28 (0.91–1.81)	0.93 (0.72–1.20)	1.00	0.97 (0.74–1.26)	1.12 (0.87–1.44)
**Cardiovascular disease**						
Number of cases (deaths)	173	192	605	730	553	729
Age-adjusted HR (95% CI)	1.45 (1.23–1.71)	1.15 (0.98–1.35)	1.06 (0.95–1.18)	1.00	1.08 (0.96–1.21)	1.17 (1.05–1.30)
Multivariable-adjusted HR (95% CI)^a^	1.33 (1.12–1.58)	1.12 (0.95–1.31)	1.05 (0.94–1.17)	1.00	1.05 (0.94–1.18)	1.11 (0.99–1.24)

^a^Adjusted for age, BMI, sleep duration, walking, exercising, smoking habit, drinking habit, education levels, occupation, perceived mental stress, marital status, and previous history of diabetes and hypertension.

When compared to the age group of 24–27 years, younger maternal ages at first delivery (<20 years) were associated with increased risks of CVD mortality in the age-adjusted analysis (Table 3). However, the risk of CVD mortality in women aged <20 years at first delivery was attenuated and became statistically non-significant after adjustment for cardiovascular risk factors. Maternal age 28–31 years at first delivery were associated with increased risks of CHD and CVD mortality, while those ≥32 years were associated with increased risk of stroke and CVD mortality. The multivariable HRs of maternal age 28–31 years at first delivery after adjustment for cardiovascular risk factors and the number of delivery were 1.29 (95% CI, 1.00–1.65) for CHD and 1.22 (95% CI, 1.10–1.36) for CVD. The multivariable HRs associated with ≥32 years at first delivery were 1.65 (95% CI, 1.26–2.16) for stroke and 1.26 (95% CI, 1.04–1.52) for CVD.

**Table 3.** Hazard ratios and 95% confidence intervals of mortality from cardiovascular disease according to maternal age at first delivery among women aged 40–79 years

	Maternal age at first delivery				

	<20	20–23	24–27	28–31	≥32
Person-years	14,225	263,184	419,219	113,694	30,178
**Stroke**					
Number of cases (deaths)	36	398	481	186	67
Age-adjusted HR (95% CI)	1.35 (0.96–1.90)	1.16 (1.01–1.32)	1.00	1.14 (0.96–1.35)	1.49 (1.16–1.93)
Multivariable-adjusted HR (95% CI)^a^	1.13 (0.80–1.59)	1.09 (0.95–1.25)	1.00	1.16 (0.98–1.38)	1.65 (1.26–2.16)
**Coronary heart disease**					
Number of cases (deaths)	17	170	211	94	19
Age-adjusted HR (95% CI)	1.43 (0.87–2.34)	1.12 (0.91–1.37)	1.00	1.29 (1.01–1.65)	0.95 (0.59–1.52)
Multivariable-adjusted HR (95% CI)^a^	1.15 (0.69–1.90)	1.04 (0.85–1.28)	1.00	1.29 (1.00–1.65)	0.91 (0.56–1.49)
**Cardiovascular disease**					
Number of cases (deaths)	88	884	1,126	476	133
Age-adjusted HR (95% CI)	1.39 (1.12–1.73)	1.10 (1.01–1.20)	1.00	1.23 (1.10–1.36)	1.25 (1.04–1.49)
Multivariable-adjusted HR (95% CI)^a^	1.19 (0.95–1.48)	1.05 (0.96–1.15)	1.00	1.22 (1.10–1.36)	1.26 (1.04–1.52)

^a^Adjusted for age, BMI, sleep duration, walking, exercising, smoking habit, drinking habit, education levels, occupation, perceived mental stress, marital status, previous history of diabetes and hypertension, and number of deliveries.

After being stratified by the number of deliveries, older maternal age at first delivery implied a 1.2- to 1.5-fold increased risks of CVD mortality in women who experienced ≥3 deliveries, compared to 24–27 years at first delivery (Table 4). The multivariable HRs of CVD for ≥28 years at first delivery were 1.42 (95% CI, 1.17–1.73) for three deliveries, 1.20 (95% CI, 0.94–1.53) for four deliveries, and 1.48 (95% CI, 1.12–1.96) for ≥5 deliveries; when the categories of ≥3 deliveries were combined the multivariable HR was 1.33 (95% CI, 1.17–1.52).

**Table 4.** Hazard ratios and 95% confidence intervals of mortality from cardiovascular disease according to maternal age at first delivery among women aged 40–79 years

	Number of deliveries: 1			Number of deliveries: 2			Number of deliveries: 3			Number of deliveries: 4			Number of deliveries: ≥5		
					
Maternal age at first delivery	<24	24–27	≥28	<24	24–27	≥28	<24	24–27	≥28	<24	24–27	≥28	<24	24–27	≥28
Person-years	9,828	24,082	26,347	89,762	174,514	64,490	94,033	142,900	33,521	39,900	41,738	9,207	32,600	21,626	3,801
**Stroke**															
Number of cases (deaths)	11	19	36	72	106	60	105	125	75	69	110	41	146	99	27
Age-adjusted HR (95% CI)	1.20 (0.57–2.53)	1.00	1.49 (0.85–2.60)	1.36 (1.01–1.84)	1.00	1.02 (0.74–1.41)	1.33 (1.03–1.73)	1.00	1.50 (1.12–2.00)	0.82 (0.61–1.11)	1.00	1.19 (0.83–1.71)	1.04 (0.81–1.35)	1.00	1.34 (0.87–2.05)
Multivariable-adjusted HR (95% CI)^a^	1.22 (0.57–2.61)	1.00	1.65 (0.93–2.91)	1.28 (0.95–1.74)	1.00	1.01 (0.73–1.39)	1.34 (1.03–1.74)	1.00	1.54 (1.15–2.06)	0.81 (0.59–1.10)	1.00	1.13 (0.78–1.64)	0.99 (0.76–1.28)	1.00	1.30 (0.84–2.01)
**Coronary heart disease**															
Number of cases (deaths)	8	15	18	20	47	24	43	48	34	36	38	15	71	47	10
Age-adjusted HR (95% CI)	0.92 (0.39–2.18)	1.00	0.85 (0.43–1.68)	0.85 (0.51–1.44)	1.00	0.87 (0.53–1.44)	1.41 (0.94–2.14)	1.00	1.85 (1.18–2.89)	1.26 (0.79–1.99)	1.00	1.24 (0.68–2.26)	1.08 (0.75–1.56)	1.00	1.00 (0.51–1.99)
Multivariable-adjusted HR (95% CI)^a^	0.71 (0.28–1.80)	1.00	0.76 (0.36–1.59)	0.77 (0.45–1.31)	1.00	0.87 (0.53–1.44)	1.44 (0.95–2.18)	1.00	1.89 (1.20–2.97)	1.19 (0.74–1.90)	1.00	1.13 (0.61–2.11)	0.98 (0.67–1.44)	1.00	0.97 (0.48–1.94)
**Cardiovascular disease**															
Number of cases (deaths)	35	60	79	145	254	159	212	291	165	176	236	95	337	219	67
Age-adjusted HR (95% CI)	1.11 (0.73–1.69)	1.00	0.97 (0.70–1.36)	1.16 (0.94–1.42)	1.00	1.07 (0.88–1.31)	1.16 (0.97–1.39)	1.00	1.42 (1.17–1.73)	1.00 (0.82–1.21)	1.00	1.25 (0.98–1.59)	1.10 (0.93–1.31)	1.00	1.47 (1.12–1.93)
Multivariable-adjusted HR (95% CI)^a^	1.06 (0.69–1.62)	1.00	1.00 (0.71–1.42)	1.09 (0.89–1.35)	1.00	1.08 (0.88–1.32)	1.19 (0.99–1.42)	1.00	1.42 (1.17–1.73)	0.98 (0.80–1.19)	1.00	1.20 (0.94–1.53)	1.03 (0.87–1.23)	1.00	1.48 (1.12–1.96)

^a^Adjusted for age, BMI, sleep duration, walking, exercising, smoking habit, drinking habit, education levels, occupation, perceived mental stress, marital status, previous history of diabetes and hypertension.

## DISCUSSION

In this prospective cohort study of Japanese women aged 40–79 years, we found a U-shaped association between the number of deliveries and risk of CVD mortality with reference to three deliveries, although the excess risk of CVD mortality associated with ≥5 deliveries was of borderline statistical significance. We also found that older maternal ages at first delivery were associated with increased risk of CVD mortality, but the young age at first delivery was not statistically significant after adjustment for cardiovascular risk factors and the number of deliveries. Furthermore, among women who experienced ≥3 deliveries, maternal age at first delivery ≥28 years was associated with increased risk of CVD mortality.

Our results were consistent with previous findings of U-shaped association between the number of deliveries and risk of CVD mortality.^4^^–^^8^ Important findings were reported by the census-based Israel Longitudinal Mortality Study II, which enrolled 62,822 women and showed that both nulliparity and higher number of deliveries were associated with increased risk of CVD mortality. The multivariable HRs of CVD mortality in women aged 45–64 years were 2.43 (95% CI, 1.49–3.96) for nulliparous and 1.64 (95% CI, 1.02–2.65) for eight deliveries.^5^ In addition, a meta-analysis of 10 cohort studies reporting 16,601 deaths, including five North American studies, the aforementioned Israeli study, and four studies from other countries (Australia, China, Korea, and Finland; one study each),^8^ indicated a U-shaped association between the number of deliveries and risk of CVD mortality, with 4 deliveries representing the nadir of risk.

The excessive risk of CVD associated with ≥5 deliveries may be attributable to increase insulin resistance and lipid abnormalities during pregnancy. A study of 42 pregnant and 24 non-pregnant Austrian women indicated that insulin levels rose after week 23 and reached the peak at week 31 of pregnancy. In addition, LDL cholesterol levels rose 2-fold from week 8 to week 36 of pregnancy, and then fell thereafter. The postpartum LDL cholesterol level in 6–8 weeks after delivery were still 50% higher than those in the non-pregnant women.^23^ Moreover, diabetes was accompanied with the larger number of delivery. In the ARIC study of 7,024 Caucasian and Africa-American women aged 45–64 years, ≥5 deliveries were 27% higher risk for diabetes than 1–2 deliveries.^24^

As for non-biological mechanisms for excessive risk of CVD mortality among nulliparous women, nulliparous women tended to have unhealthy lifestyles, such as drinking and smoking, which are well-known to be risk factors for infertility.^25^ Also, they tended to have no spouse, suggesting lower social connection, which is a risk factor for CVD.^26^

We also found an increased risk of CVD mortality with older maternal age at first delivery, especially in women with higher number of deliveries. Our result was supported by the finding from a previous study, which showed the higher age-adjusted mortality from risk of subarachnoid hemorrhage for maternal age ≥26 years at first delivery, compared to maternal age <25 years at first delivery.^18^ The exact mechanism for the association between higher maternal age at first delivery and risk of CVD mortality is unknown. However, women with higher maternal age at delivery is more likely to accompany risk of pregnancy complications, including hypertensive disorder of pregnancy and gestational diabetes,^27^ which may increase risk of CVD in later life.^28^^,^^29^

The excessive risk of CVD mortality with younger maternal age at first delivery could be explained by biological and non-biological mechanisms. Regarding the biological aspect, adolescent mothers have a larger metabolic change during pregnancy than older women.^30^^,^^31^ These metabolic changes occur during their growth phase, and the weight gained during pregnancy does not return to the baseline levels after delivery.^30^ The other reason may be related to non-biological aspects of adolescent mothers, because they are likely to have adverse socioeconomic factors, such as lower educational level and lower income in later life.^16^ Indeed, in the present study, the association between early maternal age at first delivery and risk of CVD mortality became weaker after adjustment for educational level, occupation, and marital status.

The strengths of our study are its prospective design and its large sample size, which enabled us to examine the associations between reproductive history and risk of CVD mortality with multivariable-adjustment and stratified analyses.

Some limitations need to be mentioned. First, we used a self-reported questionnaire addressing the reproductive history, for which reliability was not assessed. However, previous studies indicated an excellent agreement between the responses of two interviews about reproductive history. As for maternal age at first delivery, in which the interval between two interviews was more than two years for 28% of the participants, the kappa coefficient value was 0.94.^32^ As for the number of deliveries, the median time between the two interviews was 5 years, and the kappa coefficient value was 0.93.^33^ Second, there was no information about pregnancy complications, such as hypertensive disorders of pregnancy and diabetes, which may be associated with risk of CVD in later life. A Swedish study, with 1.4 million women, reported that a U-shaped association found between the number of deliveries and risk of CVD became weaker, but remained statically significant after further adjustment for history of pregnancy complications, including hypertension and diabetes.^3^ Third, the outcomes of the present study were mortality from stroke, CHD, and CVD, which may be biased to more advanced cases leading to deaths. However, the occurrence of premature deaths did not seem to affect our result due to the small number of CVD deaths under 60 years old (*N* = 89).

In conclusion, the number of deliveries showed a U-shaped association with the risk of total CVD mortality. Higher maternal age at first delivery was associated with increased risk of CVD, and the excessive risk found in women aged ≥28 years at first delivery was noted in women with ≥3 deliveries.
